# Paraneoplastic Autoimmunity in Thymus Tumors

**DOI:** 10.1155/1998/49484

**Published:** 1998

**Authors:** Alexander Marx, Anja Schultz, Annette Wilisch, Markus Helmreich, Regina Nenninger, Hans Konrad Müller-Hermelink

**Affiliations:** Department of Pathology University of Würzburg Josef-Schneider-Strasse 2 Würzburg D-97080 Germany

**Keywords:** Thymoma, acetylcholine receptor, titin, neurofilaments, T cells

## Abstract

Autoimmune phenomena are more frequent in thymic epithelial tumors (TET) than in any other human tumor. Mysthenia gravis (MG) is by far the most common autoimmune disease in thymoma patients. MG is characterized by muscle weakness due to autoantibodies against the acetylcholine receptor (AChR), and CD4 ^+^AChR-specific T cells play a pivotal role for the production of these autoantibodies. About 10% of MG patients have a thymoma and, interestingly, only such thymomas exhibit an MG association that maintains thymuslike morphological and functional features with respect to the homing and differentiation of immature T cells. Since AChR protein is not expressed in thymomas, the specificity of the autoimmunity in thymoma-associated MG is thought to be determined by nonreceptor proteins with AChR epitopes. Such proteins are overexpressed in cortical-type MG-associated thymomas, and medullary thymomas express these proteins at barely detectable levels. Aside from this quantitative difference, the pathogenesis of anti-AChR autoimmunity might be qualitatively different in these thymoma subtypes. Our findings suggest that an antigen-specific abnormal Tcell selection by cortical-type TET may contribute to the pathogenesis of paraneoplastic MG. In contrast, an abnormal (intratumorous) activation of autoreactive T cells may be operative in medullary thymomas.


*Developmental Immunology*, 1998, Vol. 6, pp. 129–140
Reprints available directly from the publisher
Photocopying permitted by license only

© 1998 OPA (Overseas Publishers Association)
N.V. Published by license under the
Harwood Academic Publishers imprint,
part of the Gordon and Breach Publishing Group
Printed in Malaysia

### Paraneoplastic Autoimmunity in Thymus Tumors

ALEXANDER MARX*, ANJA SCHULTZ, ANNETTE WILISCH, MARKUS HELMREICH, REGINA NENNINGER and HANS KONRAD MÜLLER-HERMELINK

*Department of Pathology, University of Würzburg, Josef-Schneider-Strasse 2, D-97080 Würzburg, Germany*

(*Revised 22 May 1997; In final form 30 May 1997*)

Autoimmune phenomena are more frequent in thymic epithelial tumors (TET) than in any other human tumor. Mysthenia gravis (MG) is by far the most common autoimmune disease in thymoma patients. MG is characterized by muscle weakness due to autoantibodies against the acetylcholine receptor (AChR), and $CD4^+$ AChR-specific T cells play a pivotal role for the production of these autoantibodies. About 10% of MG patients have a thymoma and, interestingly, only such thymomas exhibit an MG association that maintains thymuslike morphological and functional features with respect to the homing and differentiation of immature T cells. Since AChR protein is not expressed in thymomas, the specificity of the autoimmunity in thymoma-associated MG is thought to be determined by nonreceptor proteins with AChR epitopes. Such proteins are overexpressed in cortical-type MG-associated thymomas, and medullary thymomas express these proteins at barely detectable levels. Aside from this quantitative difference, the pathogenesis of anti-AChR autoimmunity might be qualitatively different in these thymoma subtypes. Our findings suggest that an antigen-specific abnormal T-cell selection,by cortical-type TET may contribute to the pathogenesis of paraneoplastic MG. In contrast, an abnormal (intratumorous) activation of autoreactive T cells may be operative in medullary thymomas.

*Keywords:* Thymoma, acetylcholine receptor, titin, neurofilaments, T cells

*Classification Categories* Abbreviations
AChR—Acetylcholine receptor, MG—myasthenia gravis, TET—thymic epithelial tumor(s)

### INTRODUCTION

Thymus tumors can be histologically divided into thymic epithelial tumors (TET, including thymomas and thymic carcinoms), lymphomas, thymic germ-cell tumors, and mesenchymal neoplasms (Müller-Hermelink et al., 1996). Strikingly, paraneoplastic autoimmunity is a feature only of TET (Müller-Hermelink, 1997). Among TET, organotypic and nonorganotypic TET can be distinguished (Kirchner et al., 1992). Organotypic TET are benign or malignant epithelial neoplasms that retain functional properties of the normal thymus with respect to the homing and maturation of immature thymocytes (Kirchner et al,

*Corresponding author.

TABLE I Frequency of MG and Expression of the AChR Epitope Alpha 373-380 in Thymic Epithelial Tumors (TET) Investigated Between 1970 and 1994 in the Department of Pathology, University of Würzburg

| Tumor | Cases investigated[a] | Frequency of MG(%) | Tumors with $\alpha$ 373-380[b] | Tumors without $\alpha$ 373-380[b] |
|---|---|---|---|---|
| **Organotypic TET** | | | | |
| Medullary thymoma | 10 | 30 | 0 | 3 |
| Mixed thymoma | 33 | 40 | 1 | 14 |
| Predominantly coritical thymoma | 15 | 40 | 6 | 5 |
| Cortical thymoma | 81 | 69 | 14 | 1 |
| Well-differentiated thymic carcinoma | 33 | 79 | 11 | 0 |
| **Nonorganotypic TET** | 24 | 0 | 2 | 1 |
| Tumors with MG | | | 30 | 8 |
| Tumors without MG | | | 4 | 16 |

[a]All cases available (frozen and paraffin).
[b]Cases with available frozen material (allowing reactivity with mAb 155 against the AChR alpha 373-380 epitope).

TABLE II Presumed Autoimmune Diseases and Immunological Systemic Disorders Observed in Association with Thymoma and MG[a]

- Pure red-cell aplasia; Hypogammaglobulinemia; white blood cells aplasia; autoimmune hemolytic anaemia
- Grave's disease (Morbus Basedow), Hashimoto thyroiditis
- Lupus erythematodes, rheumatoid arthritis, polymyositis, Sjögren's disease
- Subacute motor neuropathy
- Vitiligo, alopecia areata, bullous dermatoses
- Wegener's granulomatosis,[b] Crohn's disease, Sarcoidosis[a]

*Sources*: [a]Rosai, 1996; Marx et al., 1996ab; and Andonopoulos et al., 1991.
[b]Our own observations.

1992; Müller-Hermelink, 1996, 1997). In addition, organotypic TET are divided into medullary, mixed, predominantly cortical (organoid), and cortical thymomas and well-differentiated thymic carcinomas based on the morphological resemblance of neoplastic epithelial cells to the normal thymic epithelial-cell subtypes (Müller-Hermelink et al., 1996). The occurrence of paraneoplastic autoimmunity is restricted to organotypic TET apart from very rare exceptions (Rosai, 1996, Müller-Hermelink et al., 1996; Marx et al., 1996a). In contrast, nonorganotypic TET (squamous cell carcinomas, lympho-epitheliomalike carcinomas, clear-cell carcinomas, mucoepidermoid carcinomas, carcinoids, small-cell carcinomas, sarcomatoid carcinomas, or papillary adenocarcinomas) resemble their nonthymic counterparts in other organs and are not associated with paraneoplastic autoimmunity. This is particularly true for myasthenia gravis (MG) (Table I). Only acquired neuromyotonia, which is elicited by autoantibodies against he voltage-gated potassium channel may be an exception to this rule (Vincent et al., 1995) since it was found to be associated with thymic carcinoids that are considered nonorganotypic TET (Müller-Hermelink, 1996).

The list of autoimmune diseases associated with TET is remarkably long (Table II). While MG, hypoglobulinemias, pure red-cell aplasia, and other cytopenias occur frequently enough to suggest a pathogenetic role of the TET, this is less certain in the other autoimmune diseases in which TET are rare exceptions. Therefore, it has been suggested that thymomas may trigger either a specific defect of immune tolerance (in the case of MG and the cytopenias) or a general disturbance of immune regulation. This notion is supported by the finding that immune cytopenias and hypogammaglobulinemias

TABLE III MG Subtypes According to Thymus Pathology: Correlation with Clinical and Epidemiological Findings. MG with Neither Thymitis nor TET Exhibits an Age-Related thymus ("atrophy").

| Thymus pathology | Thymitis "Hyperplasia" | Thymoma/WDTC[a] |
|---|---|---|
| Onset of symptoms age (years) | 10-20 | 15-80 |
| Sex m:f | 1:3 | 1:1 |
| HLA association | B8; DR3 | (DR2) |
| *Autoantibodies against* | | |
| AchR | 30-80% | >90% |
| striated muscle | 10-20% | >90% |
| titin | <5% | >90% |
| neuronal structures | <10% | >90% |
| ryanodine receptor | <5% | >50% |
| Effect of thymectomy | Improvement in 80% | Deterioration in 80% |

*Sources*: See Marx et al., 1996a; Mygland et al., 1994, 1997.
[a]WDTC: well-differentiated thymic carcinoma; Kirchner et al., 1992.

almost exclusively occur in medullary thymomas whereas MG is mainly associated with cortical-type TET (Rosai, 1996; Müller-Hermelink et al., 1996).

Investigations concerning the pathogenetic links between TET and paraneoplastic autoimmunity have been hampered because of the rarity of almost all autoimmune diseases listed in Table II. This is not the case for paraneoplastic MG. Therefore, paraneoplastic MG serves as a model for the analysis of the relationship between functional properties and morphological features of the tumor, and the development of autoimmunity. In this paper, we will summarize clinical and pathological features of MG-associated TET and discuss novel findings for both the epithelial and lymphoid compartments of these neoplasms.

### RESULTS

### Clinical and Epidemiological Findings in Paraneoplastic Myasthenia Gravis

Myasthenia gravis (MG) is an autoimmune disease characterized by an autoantibody attack against the AChR at the neuromuscular junction. However, the disease is not defined by the presence of autoantibodies, but by the abnormal weakness of striated muscles after contraction and by characteristic electrophysiological features (Drachman, 1994). Except for this common clinical denominator, MG is a heterogeneous group of diseases. Since clinical and epidemiological findings exhibit a high association with characteristic thymic abnormalities, it has become common practice to subdivide MG according to the associated pathological thymic alterations (Table III).

It is significant for the pathogenesis of paraneoplastic MG that there is no age and gender predilection and almost no HLA association in paraneoplastic MG as opposed to the more frequent MG type that occurs in thymitis (Table III).

Whereas MG in thymitis patients is characterized by autoimmunity almost exclusively against the AChR, paraneoplastic MG is distinguished by a range of additional autoantibodies against striated muscle antigens (actin, myosin, actinin, ryanodine receptor, and titin) and against neuronal structures (Williams et al., 1992; Aarli et al., 1990; Marx et al., 1992; Gautel et al., 1993; Mygland et al., 1994). Finally, surgery has strikingly contrary effects on the autoimmune symptoms in MG with thymitis compared with thymoma-associated MG (Table III). These findings clearly separate paraneoplastic MG from nonparaneoplastic MG cases.

### Most Autoantigens Are not Expressed in MG-Associated Thymic Epithelial Tumors but Autoantigen Epitopes Are

To explain the unique pattern of autoimmunity against the AChR, titin, and the ryanodine receptor,

TABLE IV Expression of Autoantigen Epitopes but Not of the Main Antigens Themselves in Myasthenia Gravis-Associated Thymoma

| Autoantigen | Autoantigen expression | Expression of autoantigen epitope |
|---|---|---|
| AChR | No | Yes |
| Titin | No | Yes |
| Ryanodine receptors | No | Yes |

one has looked for the occurrence of these autoantigens in MG-associated thymomas. Although, mRNA for the AChR subunits has been detected by RT-PCR (Hara et al., 1991, 1993) and by screening of thymoma cDNA libraries (Gattenlöhner et al., 1994), AChR, titin, and ryanodine receptors have not been identified on the protein level (Kirchner et al., 1988b, Siara et al., 1991, Mygland et al., 1997, Wilisch et al., 1997). Instead, by using panels of monoclonal antibodies against these various autoantigens, single cross-reacting epitopes could be recognized with monoclonal antibodies against the AChR (Kirchner et al, 1988b; Wilisch et al., 1997), the ryanodine receptor (Mygland, 1997), and against titin (Marx, 1996a b) (Table IV). The only exception to the rule has been paraneoplastic antineuronal autoimmunity in thymoma/MG patients (Marx et al., 1992). This type of autoimmunity is partially due to autoantibodies against neurofilaments (unpublished). As shown recently, antineuronal autoimmunity is associated with the intratumorous expression not only of single epitopes but complete neurofilament proteins that are abnormal only due to their incomplete state of phosphorylation (Marx et al., 1996b).

### A Single Thymoma Molecule Contains Epitopes of Three Main Autoantiges

It has remained enigmatic until recently why thymomas in paraneoplastic MG should elicit autoimmunity against apparently unrelated molecules (AChR, titin, neuronal structures) at an almost identical frequency (Table I). However, when neurofilaments were identified in MG-associated thymomas, it became clear that the 160-kD neurofilament contains titin epitopes (Marx et al., 1996b) as well as epitopes homologous to the AChR epitope alpha 373-380 (Wilisch et al., 1997). Therefore, we suggest that the abnormal expression of a single molecule containing epitopes of the three most frequent autoantigens may be the reason for the simultaneous appearance of the three types of autoimmunity (Fig. 1). Whether neurofilaments also contain a ryanodine receptor epitope remains to be shown.

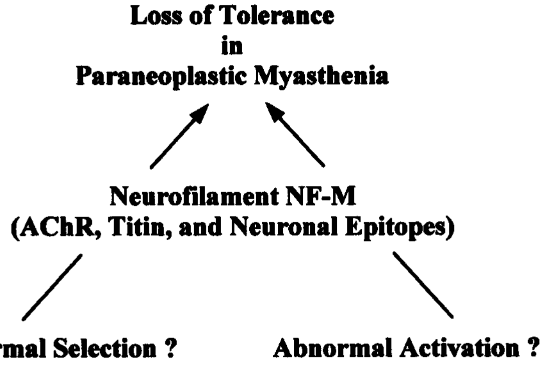

FIGURE 1 Hypotheses to explain the restricted autoimmune response against the AChR, titin, and neuronal structures regarding the fact that neither AChR nor titin are expressed in MG-associated thymic epithelial tumors. Cross-reacting epitopes of the AChR and titin occurring in the abnormally expressed 160-kD neurofilament (NFM) might elicit T-cell autoreactivity either by abnormal selection or an abnormal stimulation of T cells.

### The Expression of AChR Epitopes is Correlated with the Occurrence of MG in Thymic Epithelial Tumors

As shown in Table I, the occurrence of MG is clearly associated with the expression of the AChR alpha subunit epitope alpha 373-380 (Kirchner et al., 1988a, 1988b; Marx et al., 1996a). This association is most striking in cortical-type thymomas. In this subtype, the patients never suffer from MG when this epitope is not expressed ($n = 5$; unpublished). The absent or low expression of this AChR epitope in medullary and mixed thymomas may be one reason why paraneoplastic MG is rare in these tumors (Table I). It has been suggested that MG in these TET subtypes may be related to other so far unknown cross-reacting epitopes or a different pathogenetic mechanism (Nenninger et al., 1996).

### Increased Frequency of Autoantigen Reactive T Cells in MG-Associated Thymic Epithelial Tumors

To investigate the relevance of the abnormal neurofilament expression in MG-associated TET, we looked for the occurrence of AChR and neurofilament reactive T cells in thymic epithelial tumors. As shown in Fig. 2, T cells reacting with a recombinant neurofilament fragment and various AChR fragments were more frequently encountered in thymomas than

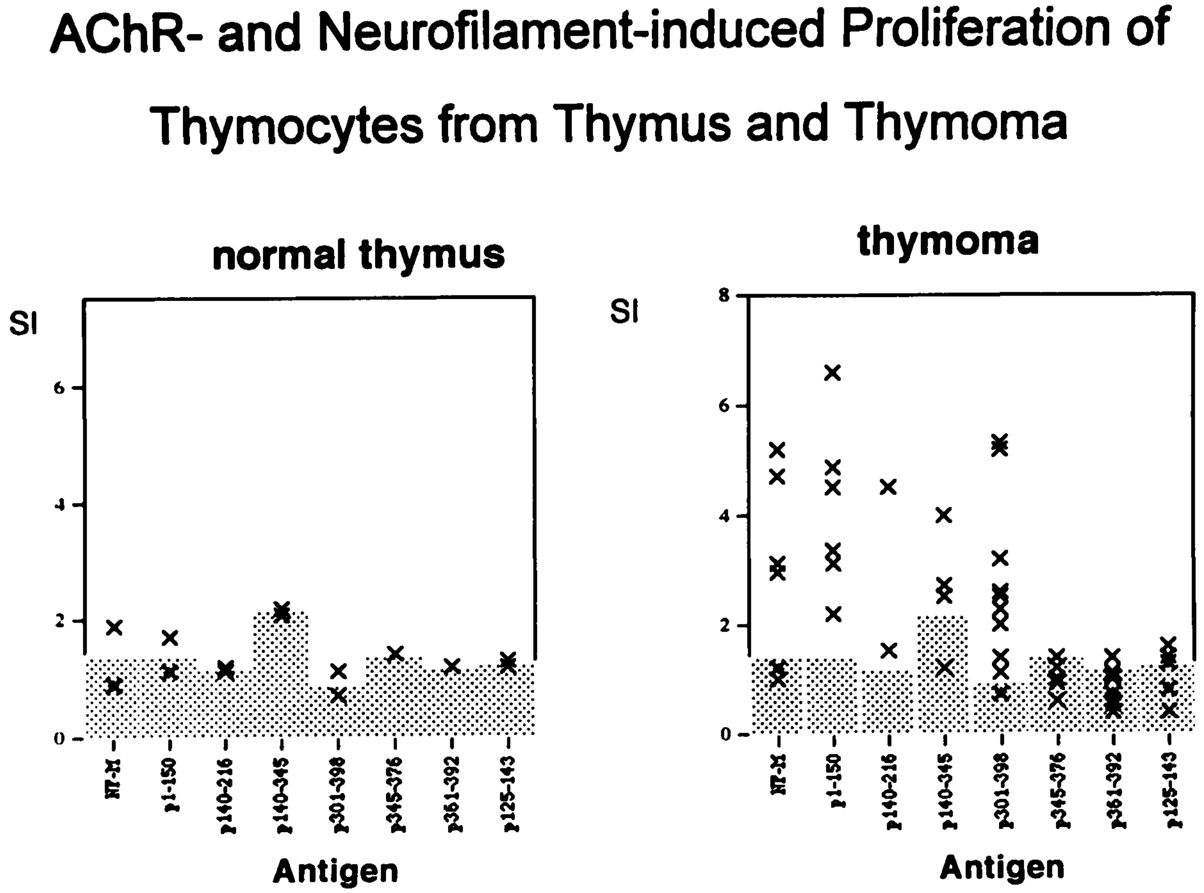

FIGURE 2 Increased frequency of AChR and neurofilament reactive T cells in thymomas. Proliferation of thymocytes from normal thymuses ($n = 6$) and mixed and cortical-type thymomas ($n = 10$) in response to the recombinant human neurofilament fragment aa459-737 (NF-M) and various recombinant fragments and peptides of the AChR alpha subunit. Proliferation was measured by $^3$H-thymidine incorporation and is given as the stimulation index (SI). Each + represents the mean of six wells per patient and antigen with $5 \times 10^4$ cells/well.

in the normal thymus. These findings are in line with those reported by Sommer et al. (1990) for AChR-reactive T cells. Like these authors, we were not able to raise T-cell responses against the AChR epitope alpha 373-380 (Fig. 2). Nevertheless, these findings show that abnormal antigen expression in thymomas may not elicit tolerance but—surprisingly—the production/accumulation of T cells reactive against these antigens.

### Abnormal Stimulation vs. Abnormal Selection Inside MG-Associated Thymic Epithelial Tumors?

It has been a major controversy whether TET elicit autoimmunity by the intratumorous stimulation of members of the normal T-cell repertoire (Sommer et al., 1990, Willcox, 1993) or an abnormal intratumorous T-cell selection is the main pathogenetic contribution of MG-associated TET (Fig. 1) (Marx et al., 1991; Müller-Hermelink et al., 1996; Wilisch et al., 1997). To substantiate the "abnormal selection hypothesis," we first confirmed that mixed and cortical thymomas and well-differentiated thymic carcinomas indeed support T-cell maturation from the earliest intrathymic precursors to the most mature T-cell subset (Nenninger et al., 1996). Furthermore, we investigated the expression of activation markers on $CD4^+/CD3^+$ mature T cells by flow cytometry. However, neither an early (ICAM-1; Fig. 3) nor a late activation antigen (CD25; not shown) could be detected in the mature $CD4^+$ T-cell compartment in over 90% of cases. This finding was confirmed when the sensitivity of the method was increased 10- to 20-fold by depleting $CD8^+$ and $CD4^+CD8^+$ cells from the TET thymocytes prior to ICAM-1 and CD25 flow cytometry (unpublished). Although it was shown previously that cortical-type neoplastic thymic epithelial cells have the abnormal capacity to present soluble antigen to mature T cells *in vitro* (Marx et al., 1994; Gilhus et al., 1995), the *ex vivo* experiments described here favor the hypothesis that thymoma tissue in the majority of cases does not stimulate intratumorous autoreactive T cells *in vivo*.

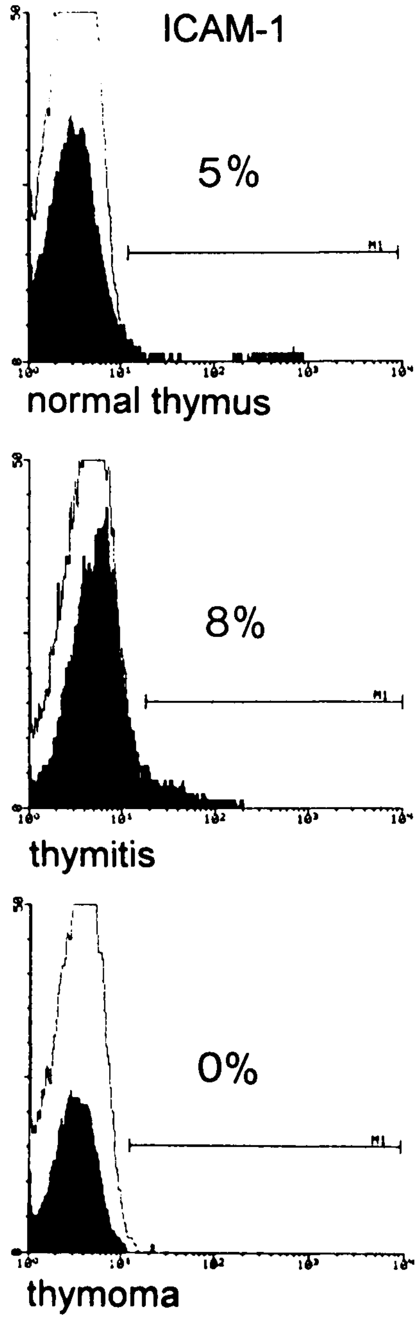

FIGURE 3 Absence of the early activation marker ICAM1 from $CD4^+/CD3^+$ T cells derived from an MG-associated cortical thymoma. For comparison, an increased number of activated T cells is detectable in thymoytes from thymitis as compared to the normal thymus. The histogram shown is representative for three other cortical thymomas and three mixed thymomas investigated in the same way by flow cytometry. A similar finding was obtained with an antibody to the late activation antigen CD25 (not shown).

### Thymomas Provide Immunosuppression in Paraneoplastic MG

TET are unique among tumors associated with paraneoplastic autoimmunity since TET surgery is followed by a deterioration of autoimmunity disease symptoms and rising autoantibody titers (Somnier, 1994; Nix et al., 1997; Schalke et al., 1997) (Fig. 4a). To investigate the biological basis of this phenomenon at a cellular level, we checked for the frequency of AChR-reactive T cells in the peripheral blood of thymoma patients at the time of surgery and 1 week later. As shown in Fig. 4b, the number of autoreactive T cells in the peripheral blood increased 1 week after surgery in thymoma patients, whereas the opposite was true in MG patients with thymitis. These findings show that MG-associated TET exert an immunosuppressive effect not only with respect to autoantibodies (Fig. 5a), but also against antigen-specific T cells (Fig. 4b). The longer-term fate of patients following surgery with respect to the levels of autoreactive T cells in the blood has yet to be evaluated.

### DISCUSSION

The key features of MG-associated cortical type TET are as follows: (1) The maintenance of thymuslike functional properties with respect to the homing and maturation of immature T cells. (2) The abnormal expression of cross-reacting AChR, titin, ryanodine receptor, and neuronal epitopes by the neoplastic epithelial cells in a cortical-type microenvironment. (3) The increased number of intratumorous autoreactive T cells against these autoantigens (as shown for the AChR and neurofilaments). (4) The apparent absence of activated intratumorous $CD4^+$ T cells.

The latter two features taken together suggest that the increased number of autoreactive intratumorous T cells results mainly from the preferential intratumorous production/selection of autoreactive T cells (Marx et al., 1991). As an alternative possibility, enrichment of MG-associated TET in autoaggressive T cells by T cell recirculation has been suggested (Sommer et al., 1990). In fact, recirculation of mature T cells into nonneoplastic thymic tissue in various species is preferentially achieved by activated but much less so by naive T cells (Surh et al., 1993). Nevertheless, we consider this an unlikely possibility to explain the high number of autoaggressive T cells in thymomas. In support of this hypothesis and using tetanus-toxin-specific T-cell proliferation as readout, we found recently that mature T cells recirculate regularly into the normal thymus (including residual thymic tissue adjacent to thymomas) but almost never into thymomas (unpublished).

Considering the high association between abnormally expressed autoantigen epitopes (Table I) in MG-associated TET, we further suggest that the increased production of antigen-specific T cells might result from the presentation of cross-reacting peptides in MHC class II molecules on neoplastic thymic epithelial cells. Obviously, such abnormally selected autoreactive T cells have to be exported to the extratumorous lymphoid tissues in order to become activated and provide help to autoaggressive B cells producing autoantibodies.

In our opinion, all the features outlined so far can best be reconciled with a two-stage model, as given in Fig. 5. According to this model, the MG-associated TET provide an increased number of autoreactive naive T cells, and on the second stage, these T cells are activated in the extratumorous "periphery." The "periphery" can be clearly the residual thymus close to the TET since we could detect a high number of antigen-specific T cells there (unpublished). However, other lymphoid organs and probably the bone marrow also have to play a role in this process, because extended thymomectomy (including the residual thymus) is not followed by a decline but by an increase of autoantibody titers instead (Fig. 4a). It has not been elucidated which autoantigen(s) are maintaining this prolonged autoimmune reaction although the AChR itself is an obvious candidate. Regardless whether one favors the "abnormal selection" or "abnormal stimulation" hypothesis, it has remained enigmatic how the intrathymomatous expression of single cross-reacting epitopes of the AChR, titin, and the ryanodine receptor might elicit (or stimulate) intratumorous antigen-specific T cells

**Thymoma-mediated Immunosuppression in Paraneoplastic MG**

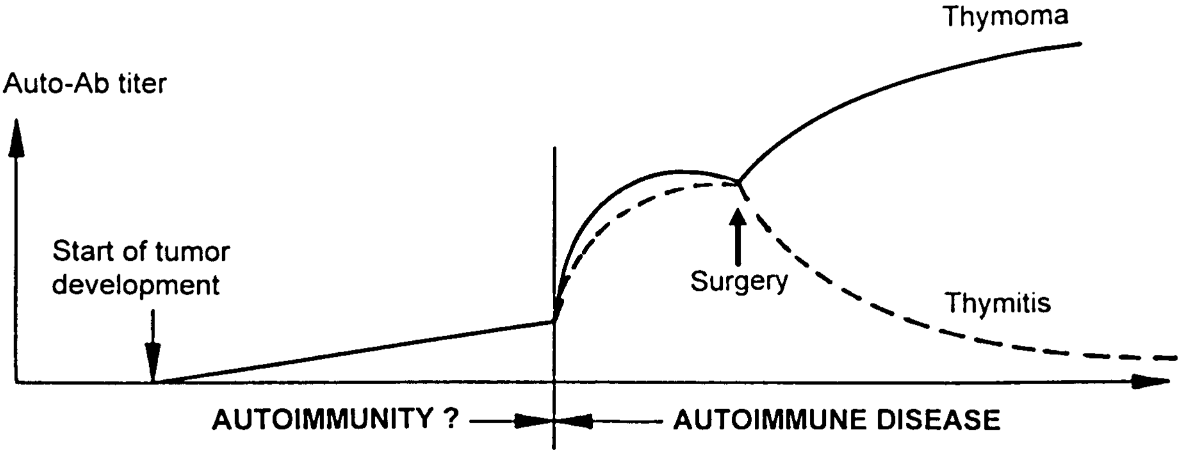

a) Immunosuppressive Effects on Autoantibodies b) Immunosuppressive Effects on AChR- and NF-reactive T cells

Percentage of positive T cell proliferation assays

| | immediately after surgery | 7 days after surgery |
|---|---|---|
| Thymoma | 29% | 49% |
| Thymitis | 34% | 29% |

FIGURE 4 (a) Opposite effect of thymectomy and tumor surgery on the autoantibody titer in patients with MG-associated thymitis (dashed curve) and thymoma (solid curve). The rise in autoantibody titer is usually accompanied by deterioration of the clinical state (Somnier, 1994; Nix et al., 1997; Schalke et al., 1997). (b) Increased frequency of AChR and neurofilament-reactive T cells in the peripheral blood 1 week after surgery in MG patients with mixed and cortical thymoma ($n = 9$). The figures given indicate the percentage of positive wells detected in antigen-specific T-cell proliferation assays, combining all assays with the stimulating autoantigen fragments given in Fig. 2. By contrast, the frequency of autoreactive T cells in patients with thymitis declines slightly after surgery.

that we (Fig. 2) and others (Sommer et al., 1990) have shown to recognize a broad spectrum of autoantigen epitopes (including cytoplasmic sequences).

Future experiments will have to show whether further cross-reacting epitopes occur inside TET. Another apparent paradox is the finding that expression of the AChR epitope alpha 373-380 in TET is clearly associated with anti-AChR autoimmunity, but that AChR alpha 373-380 peptide-specific T cells have not been detected in MG/thymoma patients (Fig. 2). According to the pathogenetic model suggested in Fig. 5, we favor the view that the peptide homologues of alpha 373-380 in neoplastic epithelial cells could function as selecting peptides for *immature* thymocytes (Marx et al., 1991). In this case, one would in fact predict that *mature* T cells might not respond to these abnormally expressed peptides because selecting peptides in the thymus is known to be nonstimulatory or even antagonistic for mature T cells outside the thymus (Ashton-Rickardt and Tonegawa, 1994; Hogguist et al., 1994). This hypothesis is presently under investigation.

The pathogenesis of paraneoplastic MG in *medullary* thymomas might be different from that in cortical-type TET given the fact that abnormally expressed, cross-reacting autoantigen epitopes have not been detected in the former so far (Marx et al., 1996a; Müller-Hermelink et al., 1996). In addition, we found a substantial number of ICAM-1 and CD25-positive, activated T cells by FACS analysis in a case

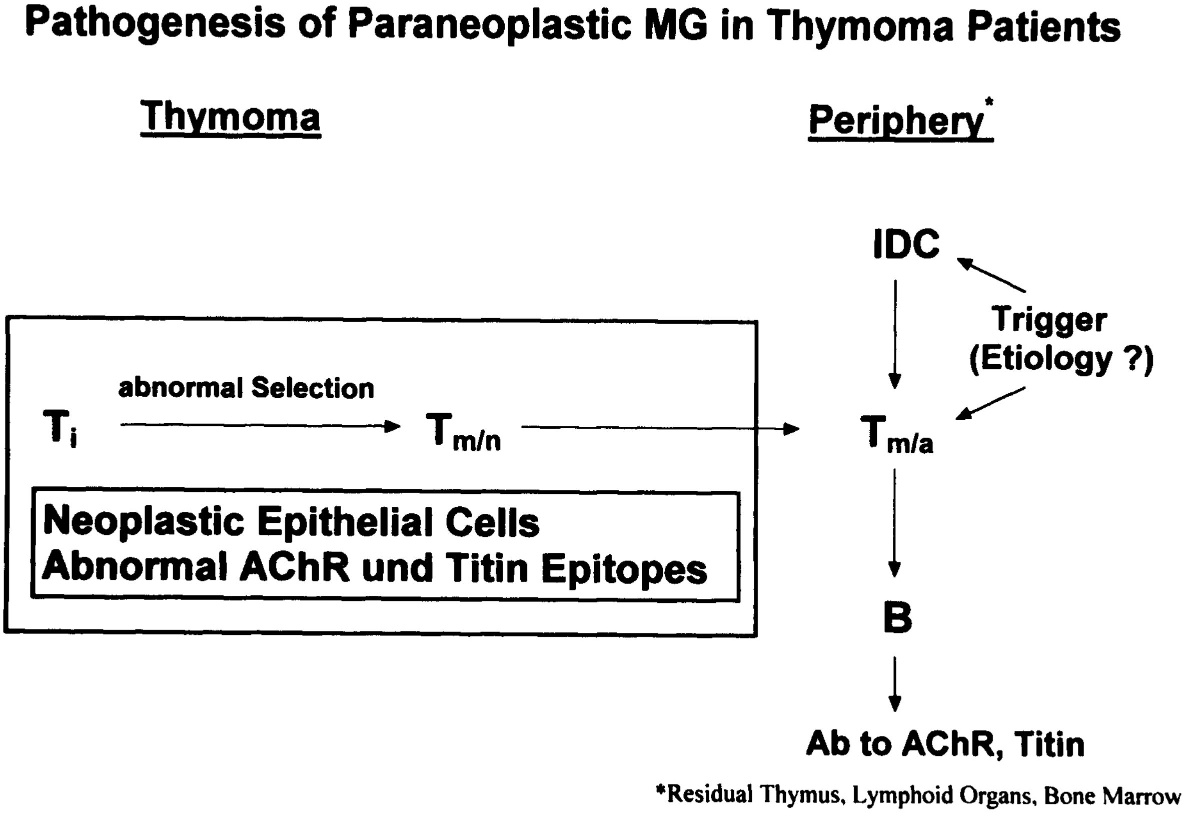

FIGURE 5 Pathogenesis of paraneoplastic MG in thymoma patients. According to this "two-step model," abnormal AChR and titin epitopes provide the molecular basis for an abnormal intratumorous selection that turns immature T cells ($T_i$) into mature and native T cells ($T_{m/n}$). In a second step, these AChR and titin-specific T cells are thought to leave the thymus and become actived T cells ($T_{m/a}$) only outside the tumor. The etiology-triggering T-cell activation is unknown (Vincent, 1994).

of MG-associated medullary thymoma (Nenninger et al., 1996). Whether these findings indeed indicate an intratumorous activation of autoantigen-specific T cells has to be substantiated by functional investigations in a larger series of medullary thymomas.

Another major finding in our studies is the detection of an immunosuppressive effect of TET on AChR and neurofilament reactive T cells in patients with paraneoplastic MG. As shown in Fig. 4, the clinical deterioration of MG symptoms and autoantibody titers is accompanied by an augmented antigen-specific T-cell response. Whether this immunosuppressive effect of TET is exerted by soluble factors (e.g., IL-10 or TGF-$\beta$) or by antigen-specific suppressor T cells has not definitely been shown. However, we have preliminary evidence that antigen-specific suppressive mechanisms are indeed effective, which has important implications for future immunotherapeutic strategies.

### MATERIALS AND METHODS

### Patients and Tissues

The clinical data of patients with paraneoplastic MG and the histological findings of their TET summarized in this paper have been published previously (Kirchner et al., 1992; Müller-Hermelink et al., 1996; Marx et al., 1996a; Nenninger et al., 1996). Normal thymuses were obtained during thoracic surgery. Tumors were classified according to Müller-Hermelink and co-workers (1996, 1997). The diagnosis of generalized MG was based on clinical findings, electrophysiological investigations, and anti-AChR serum titers that were >1 nmol/l.

### Immunohistochemistry

The application of the anti-AChR monoclonal antibody (mAb) 155 and of the various anti-neurofilament and anti-titin mAb in a three-step immunoperoxidase staining has been described previously (Kirchner et al., 1988b; 1992; Marx et al., 1996b).

### Biochemical and Molecular Biological Techniques

The detection of neurofilament mRNA by RT-PCR and of neurofilament proteins by Western blotting were described recently (Marx et al., 1996b). Detection of titin and AChR epitopes in neurofilaments was achieved using recombinantly produced neurofilament fragment NF-M aa459-737 for Western blotting applying mAb 155 and the anti-titin mAb 63/15 (Wilisch et al., 1997).

### Flow Cytometry

Three-color FACS analyses on a Becton-Dickinson FACS scan was performed as described previously (Nenninger et al., 1996).

### T-Cell Proliferation Assay

The T-cell proliferation assay using density-gradient-enriched cell suspensions from minced thymoma or nonneoplastic thymic tissue or peripheral blood mononuclear cells were performed as described by Melms et al. (1992). Since density-gradient-enriched thymoma-cell suspensions (and to a smaller extent thymus-cell suspensions) were found to exhibit a suboptimal antigen-presenting capacity, $5 \times 10^4$ responder cells from thymoma and thymus samples were supplemented with $10^5$ irradiated (50 Gy) peripheral blood mononuclear cells for improved antigen presentation (unpublished). Antigens used in proliferation assays were supplied at optimal concentrations (peptides, 10 $\mu$g/ml; recombinant protein fragments, 1 $\mu$g/ml).

### *Acknowledgments*

We thank Mrs. Ch. Kohaut, Mrs. E. Oswald, Mrs. A. Homburger, and Mr. E. Schmitt for expert technical assistance and Mrs. B. Göbel for secretarial help. The paper was supported by the DFG (grant Ma 1484/2-1), the BMBF (Project C-5, KFZ Würzburg), and the BIOMED Project of the European Community.

### References

Aarli J.A., Stefansson K., Marton L.S.G. and Wollmann R.L. (1990). Patients with myasthenia gravis and thymoma have in their sera IgG autoantibodies against titin. Clin. Exp. Immunol., **82**: 284-288.

Andonopoulos A.P., Papthanasopoulos P.G., Karatza C. and Angelopoulos J. (1991). Sarcoidosis in a patient with myasthenia gravis. Case report and review of the literature. Clin. Rheumatol. **10**: 323-325.

Ashton-Rickardt P.G. and Tonegawa S. (1994). A differential-avidity model for T cell selection. Immunol. Today **15**: 362-366.

Drachman D.B. (1994). Myasthenia gravis. N. Engl. J. Med. **330**: 1797-1810.

Gattenlöhner S., Brabletz T., Schultz A., Marx A., Müller-Hermelink H.K. and Kirchner Th. (1994). Cloning of a cDNA coding for the acetylcholine receptor alpha-subunit from a thymoma associated with myasthenia gravis. Thymus **23**: 103-113.

Gautel M., Lakey A., Barlow D.P., Holmes Z., Scales S., Leonard K., Labeit S., Mygland A., Gilhus N.E. and Aarli J.A. (1993). Titin antibodies in myasthenia gravis: Identification of a major immunogenic-region of titin. Neurology **43**: 1581-1585.

Geuder K.I., Marx A., Witzemann V., Schalke B., Kirchner Th. and Müller-Hermelink H.K. (1992). Genomic organization and lack of transcription of the nicotinic receptor subunit geneses in myasthenia gravis associated thymoma. Lab. Invest. **66**: 452-458.

Gilhus N.E., Willcox N., Harcourt G., Nagvekar N., Beeson D., Vincent A. and Newsom-Davis J. (1995). Antigen presentation by thymoma epithelial cells from myasthenia gravis patients to potentially pathogenic T cells. J. Neuroimmunol. **56**: 65-76.

Hara Y., Hayashi K., Ohta K., Itoh N. and Ohta M. (1993). Nicotinic acethylcholine receptor mRNAs in myasthenia thymuses. Association with intrathymic pathogenesis of myasthenia gravis. Biochem. Biophys. Res. Commun. **194**: 1269-1275.

Hogguist K.A., Jameson S.C., Heath W.R., Howard J.L., Bevan M.J. and Carbone F.R. (1994). T cell receptor antagonist peptides induce positive selection. Cell **76**: 17-27.

Kirchner Th., Hoppe F., Schalke B. and Müller-Hermelink H.K. (1988a). Microenvironment of thymic myoid cells in myasthenia gravis. Virchows Archiv. B. Cell. Pathol. **54**: 295-302.

Kirchner Th., Schalke B., Buchwald J., Ritter M., Marx A. and Müller-Hermelink H.K. (1992). Well-differentiated thymic carcinoma. An organotypic low-grade carcinoma with relationship to cortical thymoma. Amer. J. Surg. Pathol. **16**: 1153-1169.

Kirchner T., Tzartos S., Hoppe F., Schalke B., Wekerle H. and Müller-Hermelink H.K. (1988b). Pathogenesis of myasthenia gravis. Acetylcholine receptor-related antigenic determinants in tumor-free thymuses and thymic epithelial tumors. Amer. J. Pathol. **130**: 268-280.

Lehrmann P.V., Sercarz E.E., Firsthuber T., Dayan C.M. and Gammon G. (1993). Determinant spreading and the dynamics of the autoimmune T cell repertoire. Immunol. Today **14**: 203-208.

Marx A., Geuder K.I., Schoepfer R., Tzartos S., Kristoffersson U., Schalke B., Kirchner Th. and Müller-Hermelink H.K. (1991). Analysis of the acetyicholine receptor epitope-bearing protein p153 in thymomas favours "false-positive T cell selection" as a mechanism of paraneoplastic myasthenia gravis. In Lymphatic Tissues and In Vivo Immune Responses (New York: Marcel Decker), pp. 577-583.

Marx A., Kirchner Th., Greiner A., Müller-Hermelink H.K., Schalke B. and Osborn M. (1992). Neurofilament epitopes in thymoma and antiaxonal autoantibodies in myasthenia gravis. Lancet **339**: 707-708.

Marx A., O'Connor R., Geuder K.I., Hoppe F., Schalke B., Tzartos S., Kalies I., Kirchner Th. and Müller-Hermelink H. K. (1990). Characterization of a protein with an acetylcholine receptor epitope from myasthenia gravis-associated thymomas. Lab. Invest. **62**: 279-286.

Marx A., Schömig D., Schultz A., Gattenlöhner S., Jung A., Kirchner T., Melms A. and Müller-Hermelink H.K. (1994). Distribution of molecules mediating thymocyte-stroma interactions in human thymus, thymitis and thymic epithelial tumors. Thymus **23**: 83-93.

Marx A., Schultz A., Wilisch A., Nenninger R. and Müller-Hermelink H.K. (1996a). Myasthenia gravis. Verhandl. Dtsch. Ges. Pathol. **80**: 116-120.

Marx A., Wilisch A., Schultz A., Greiner A., Magi B., Pallini V., Schalke B., Toyka K.V., Kirchner Th. and Müller-Hermelink H.K. (1996b). Expression of neurofilaments and of a titin epitope in thymic epithelial tumors. Amer. J. Pathol. **148**: 1839-1850.

Melms A. Malcherek G., Gern U., Wiethölter H., Müller C.A., Schoepfer R. and Lindstrom J. (1992). T cells from normal and myasthenic individuals recognize the human acetylcholine receptor: Hoeterogeneity of antigenic sites on the alpha-subunit. Ann. Neurol. **31**: 311-318.

Müller-Hermelink H.K., Marx A. and Kirchner T. (1996). Thymus and mediastinum. In Anerson's Pathology, 10th ed., Damjanov I. and Lindner J., Eds. (St. Louis: Mosby Year-Book Inc.), pp. 1218-1255.

Müller-Hermelink H.K., Wilisch A., Schultz A. and Marx A. (1997). Characterization of the human thymic microenvironment: Lymphoepithelial interaction in normal thymus and thymoma. Arch. Histol. Cytol. **60**: 9-28.

Mygland A., Aarli J.A., Matre R. and Gilhus N.E. (1994). Ryanodine receptor antibodies related to severity of thymoma associated myasthenia gravis. J. Neurol. Neurosurg. Psych. **75**: 843-846.

Mygland A., Kuwajima G., Mikoshiba K., Aarli J.A. and Gilhus N.E. (1997). Thymomas express ryanodine receptor epitopes. In Epithelial Tumors of the Thymus (Pathology, Biology, Treatment), Marx A. and Müller-Hermelink H.K., Eds., (London: Plenum Press), pp.

Nenninger R., Schultz A., Wilisch A., Müller-Hermelink H.K. and Marx A. (1996). Abnormal T cell maturation in myasthenia gravis-associated thymomas. Verh. Dtsch. Ges. Path. **80**: 256-260.

Nix W.A., Große-Höötmann H., Kirchner T. and Marx A. (1997). Management of thymectomized myasthenic patients. In Epithelial Tumors of the Thymus (Pathology, Biology, Treatment), Marx A. and Müller-Hermelink H.K., Eds., (London: Plenum Press), pp. 351-356.

Rosai J., Ed. (1996). Mediastinum. In Ackermann's Surgical Pathology, 8th ed., (St. Louis: Mosby Year-Book Inc.), pp. 1:435-491.

Schalke B.C.G., Schmitt I., Marx A., Toyka K.V. and Müller-Hermelink H.K. (1997). Long-term prognosis of patients with thymoma-associated myasthenia gravis. In Epithelial Tumors of the Thymus (Pathology, Biology, Treatment), Marx A. and Müller-Hermelink H.K., eds. (London: Plenum Press), pp. 329-335.

Siara J., Rüdel R. and Marx A. (1991). Absence of acetylcholine-induced current in epithelial cells from thymus glands and thymomas of myasthenia gravis patients. Neurology **41**: 128-131.

Sommer N., Willcox N., Harcourt G.C. and Newsom-Davis J. (1990). Myasthenic thymus and thymoma are selectively enriched in acetylcholine receptor-reactive T cells. Ann. Neurol. **28**: 312-319.

Somnier F.E. (1994). Exacerbation of myasthenia gravis after removal of thymomas. Acta Neurol. Scand. **90**: 56-66.

Surh C.D., Sprent J. and Webb S.R. (1993). Exclusion of circulating T cells from the thymus does not apply in the neonatal period. J. Exp. Med. **177**: 379-385.

Vincent A. (1994). Aetiological factors in development of myasthenia gravis. Adv. Neuroimmunol. **4**: 355-371.

Vincent A., Roberts M., Willison H., Lang B. and Newsom-Davis J. (1995). Autoantibodies, neurotoxins and the nervous system. J. Physiol. Paris **89**: 129-136.

Vincent A. and Willcox N. (1994). Characterization of AChR specific T cells in myasthenia gravis. Immunol. Today **15**: 41-42.

Wilisch A., Schultz A., Jung A., Schalke B., Toyka K.V., Pallini V., Müller-Hermelink H.K. and Marx A. (1997). Titin epitope in thymoma. In Epithelial Tumors of the Thymus (Pathology, Biology, Treatment), Marx A. and Müller-Hermelink H.K., eds. (London: Plenum Press), pp. 221-227.

Willcox N. (1993). Myasthenia gravis. Cur. Opin. Immunol. **5**: 910-917.

Williams C.L., Hay J.E., Hiatt T.W. and Lennon V.A. (1992). Paraneoplastic IgG Striational Autoantibodies Produced by Clonal Thymic B Cells and in Serum of Patients with Myasthenia Gravis and Thymoma React with Titin. Lab. Invest. **66**: 331-336.

